# Real-time inversion recovery for infarct visualization during MR-guided interventions

**DOI:** 10.1186/1532-429X-18-S1-P205

**Published:** 2016-01-27

**Authors:** Adrienne E Campbell-Washburn, Toby Rogers, Jonathan R Mazal, Michael S Hansen, Robert J Lederman, Anthony Z Faranesh

**Affiliations:** Cardiovascular and Pulmonary Branch, Division of Intramural Research, National Heart, Lung, and Blood Institute, National Institutes of Health, Bethesda, MD USA

## Background

Real-time MR imaging is appealing for dynamic procedural guidance. Some MRI-guided procedures, such as catheter ablation or myocardial biopsy, may require the interventionist to visualize infarcted tissue in real-time in order to navigate devices relative to the lesion. Methods to interleave late gadolinium enhancement (LGE) images into a real-time imaging stream (1 in 5 frames) have been described previously for infarct visualization (1). Here, we implemented a real-time inversion recovery sequence to provide a stream of LGE images for procedural guidance, designed to rapidly toggle between high-frame rate "navigation mode" and lower-frame rate "infarct visualization mode".

## Methods

Gadopenetate dimeglumine (Bayer Healthcare, Wayne, New Jersey) was administered intravenously (0.2 mmol/kg) to a swine model of myocardial infarction. Standard real-time imaging was used for "navigation mode" (bSSFP, TE/TR = 1.27/2.54 ms, flip angle = 45°, FOV = 300 mm, slice thickness = 6 mm, matrix = 192 x 144, GRAPPA factor = 2). In "infarct visualization mode", a non-selective inversion pre-pulse was performed before each real-time bSSFP acquisition. Inversion time (TI) was implemented as a real-time interactive parameter to enable optimal myocardial nulling, typical TI = 320 ms (approximately 25 minutes post-injection). The next inversion pulse immediately followed the image acquisition, with no additional time for signal recovery. A checkbox was used to switch between imaging modes on the fly.

## Results

**Figure 1:**
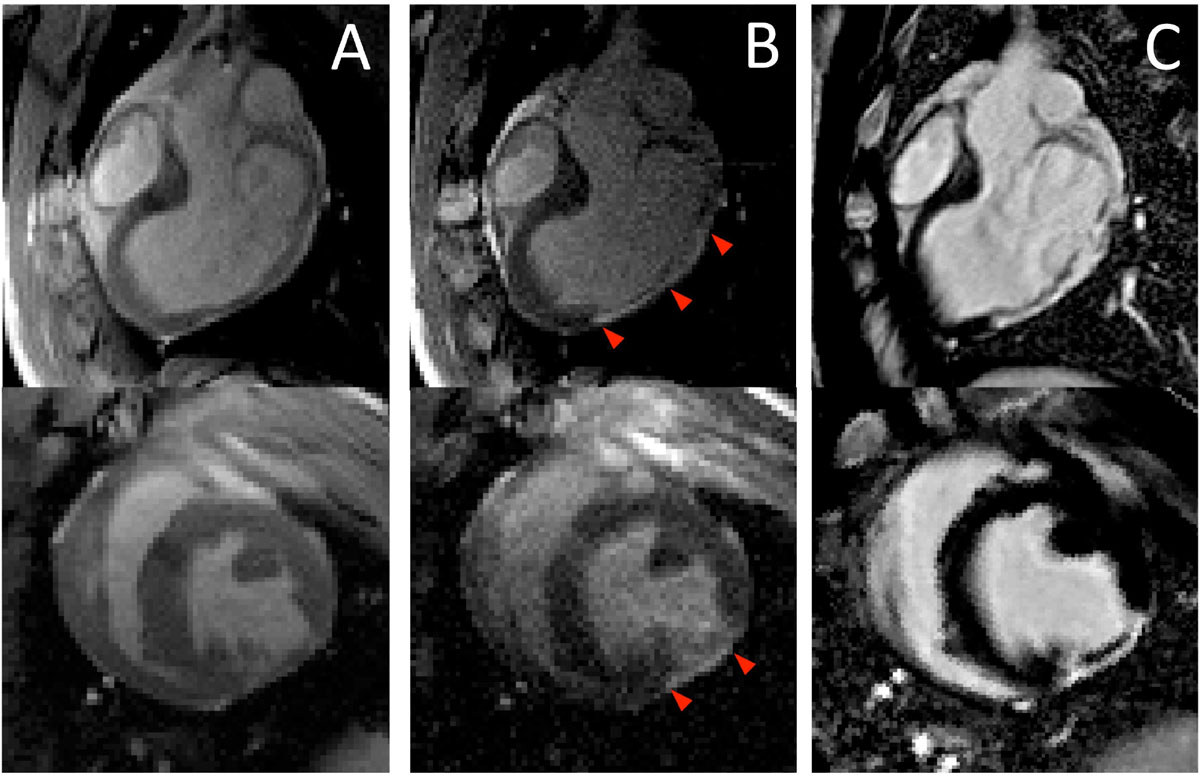
**Comparison of standard real-time bSSFP images ("navigation mode") (A), real-time inversion recovery images ("infarct visualization mode") (B) and high-resolution ECG-gated breath-held LGE images (C) in two planes**. Red arrowheads (B) indicate improved real-time infarct visualization compared to standard real-time imaging (A), and accurate infarct delieation compared to gold-standard late gadolinium enhancement images (C).

"Navigation mode" provided a temporal resolution of 244 ms/frame (4.1 frames/s), and was used for coarse navigation of devices requiring high frame-rate imaging. "Infarct visualization mode" provided a stream of myocardium-nulled LGE images with a temporal resolution of 444 ms/frame (2.3 frames/s) and was used for improved infarct delineation during fine device navigation relative to infarct location. Figure 1 demonstrates the real-time infarct visualization with a comparison to a high-resolution ECG-gated breath-held LGE images (2).

## Conclusions

This imaging method provides real-time stream of LGE images that can be used to navigate devices relative to infarcted tissue, with only a small penalty in frame-rate. This sequence was designed with added flexibility to toggle between high frame-rate imaging and lower frame rate infarct visualization. This method will be useful for MRI-guided procedures such as catheter ablation and myocardial biopsy.
